# ﻿A new remarkable *Vanilla* Mill. (Orchidaceae) species endemic to the Espinhaço Range, Brazil: its phylogenetic position and evolutionary relationships among Neotropical congeners

**DOI:** 10.3897/phytokeys.227.101963

**Published:** 2023-06-08

**Authors:** Emerson Ricardo Pansarin, Euler da Luz Fernandes Menezes

**Affiliations:** 1 Department of Biology, Faculty of Philosophy, Sciences and Literature of Ribeirão Preto, University of São Paulo, Av. Bandeirantes 3900, Ribeirão Preto, SP, 14040-901, Brazil University of São Paulo Sao Paolo Brazil; 2 Postgraduate Program of Graduation in Forest Science, Federal University of Vales do Jequitinhonha e Mucuri (UFVJM), Highway MGT 367-km 583, n° 5.000, Alto da Jacuba, Diamantina, MG, 39.100-000, Brazil Federal University of Vales do Jequitinhonha e Mucuri Diamantina Brazil

**Keywords:** Atlantic forest, Brazilian campos rupestres, Cerrado vegetation, evolution, molecular phylogeny, Neotropics, orchids, Vanilleae, Vanilloideae

## Abstract

During surveys conducted on Neotropical *Vanilla*, a new endemic species was found in the Brazilian campos rupestres of the Espinhaço Range. Here, this new remarkable *Vanilla* species, namely *V.rupicola* Pansarin & E.L.F. Menezes, is described and illustrated. A phylogeny for *Vanilla* is presented and the relationships between Neotropical species are discussed. The position of *V.rupicola* among Neotropical *Vanilla* is discussed within an evolutionary context. *Vanillarupicola* is recognized by its rupicolous habit, its reptant stems, and its sessile and rounded leaves. This remarkable new taxon emerges in a clade that includes *V.appendiculata* Rolfe and *V.hartii* Rolfe. Vegetative and floral features support a close relationship between *V.rupicola* and sister taxa, mainly regarding the apical inflorescence (*V.appendiculata*), the type of appendages of the central crest of the labellum, and the labellar color pattern. Phylogenetic inference suggests that the circumscription of Neotropical *Vanilla* groups needs revision.

## ﻿Introduction

An endemism area is defined as a geographic region that contains species that do not occur anywhere else (e.g. Platnick 1991). Among the Brazilian biodiversity hotspots, the Espinhaço Range of Minas Gerais (ERMG), situated between the Atlantic Forest and Cerrado Biomes, shows one of the richest floras in Brazil (Myers et al. 2000), with a high frequency of endemisms (Hensold 1988; Rapini et al. 2002; Versieux and Wendt 2007). Orchidaceae Juss. is one of the most diverse and important plant families in ERMG, with many endemic taxa having been described (Barros 1987; Barros and Pinheiro 2004; Azevedo and Van Den Berg 2007; Batista et al. 2016; Salazar et al. 2019), including members of the Vanilloideae Szlach. (Pansarin 2004).

Vanilloideae is divided into two tribes: Pogonieae Pfitzer ex Garay & Dunst. and Vanilleae Blume. With more than 100 species distributed throughout tropical regions of Asia, Africa and Americas, *Vanilla* Mill. is the most species-rich genus among Vanilleae (e.g. Cameron 2003). *Vanilla* is monophyletic (*Dictyophyllaria* Garay included; Pansarin 2010; Pansarin et al. 2012) with three well-supported clades, two of which occur throughout the Neotropics (Bouetard et al. 2010; Pansarin and Ferreira 2022). Among *Vanilla*, a Neotropical clade with thin and reticulate-veined leaves emerges as a sister to a clade that includes two subclades, an African/Asian/Caribbean group and a second, strictly Neotropical one. In the latter, the epiphyte and bird-pollinated *V.palmarum* (Salzm. ex Lindl.) Lindl. is positioned as a sister to a hemi-epiphytic clade whose species are almost completely pollinated by euglossine bees (Pansarin and Ferreira 2022).

Besides showing high diversity and ecological importance (Pansarin and Pansarin 2014; Pansarin et al. 2014), *Vanilla* is also the most economically important genus among the orchids since the fruits of the Neotropical *V.planifolia* Andrews and their relatives are a source of vanillin flavor. Efforts are currently underway to determine the diversity and the natural history of Neotropical *Vanilla* (Pansarin 2019, 2022; Pansarin and Ferreira 2022; Pansarin and Suetsugu 2022), and its potential use for gastronomy and industry (e.g. Oliveira et al. 2022). However, there is still much to know since new species have been found and described, notably in the Brazilian flora (e.g. Pansarin and Miranda 2016; Fraga et al. 2017).

In the course of the developing studies on Neotropical *Vanilla*, a new species has been found in Brazilian campos rupestres. A taxonomic description of this new remarkable *Vanilla* is presented here and a morphological comparison with other Neotropical taxa is provided. The phylogenetic position of *V.rupicola* within *Vanilla* and the relationships among Neotropical groups are discussed based on a molecular phylogeny for the genus.

## ﻿Materials and methods

The fieldwork was performed in the municipality of Diamantina (approx. 18°14'17"S, 43°36'40"W; 1,288 m elevation) in the state of Minas Gerais, Southeastern Brazil. The Diamantina Plateau is situated in the southern region of the Espinhaço Range, at the boundaries of Serra do Cipó and Grão Mogol (Gonçalves et al. 2017). It is included in the Espinhaço Range Biosphere Reserve (UNESCO 2005), located between the Atlantic Forest and the Cerrado domains, two of the main global biodiversity hotspots in the world (Myers et al. 2000). The climate of the region is classified as “Cwb”, namely subtropical highland climate with dry winters (Köppen 1948).

Fresh and herbarium material of flowering and fruiting plants was used for the study. Photographs were based on specimens collected in the field. Measurements were made directly on floral structures using a Vernier Caliper. The vegetative structures, inflorescence and flowers were photographed with a Nikon D-SLR D800 camera and a Micro Nikkor 105 mm f2.8 lens. Floral details were analyzed with a Stereozoom Leica S8 APO stereomicroscope with an integrated photo output. Digitized images were used for diagramming a template over a black background, following the model presented by Hoehne (1945), using a Microsoft PowerPoint software. The final template was exported as a 600 dpi .TIFF file.

The terminology for describing shapes followed Radford et al. (1974). Features specific to Orchidaceae were based on Dressler (1993) and Pridgeon et al. (1999). The infrageneric classification of Neotropical *Vanilla* groups followed Soto-Arenas and Cribb (2010). The original description and digital images from holotypes of related species of *Vanilla* were consulted. Plant specimens were vouchered according to usual techniques (Fidalgo and Bononi 1989) and then deposited in the BHCB, DIAM, HDJF, LBMBP, SPFR, SPF and UEC herbaria, which were also examined in order to study the Brazilian diversity of *Vanilla.* Additional living specimens were collected in the field and are under cultivation at the *Vanilla* germplasm bank from the LBMBP Orchid House (Orchidarium of the Laboratory of Molecular Biology and Systematics of Plants), University of São Paulo (FFCLRP-USP), municipality of Ribeirão Preto (approx. 21°10′S, 47°48′W; 546 m a.s.l.), state of São Paulo, Brazil, available at https://www.lbmbplab.net/vanillacollection.

The conservation status of the new taxon was defined according to the IUCN red list categories and criteria and guidelines (IUCN 2012, 2016).

### ﻿Taxon sampling for phylogenetic analysis

A total of 42 *Vanilla* accessions (37 species) were analyzed and are referred to here as the ingroup, which represents an increase of 10 *Vanilla* species (16 new accessions) over previous investigations (Pansarin and Ferreira 2022). *Lecanorchismultiflora* J.J. Sm. was selected as an outgroup according to previous phylogenetic studies on Vanilloideae (e.g. Pansarin and Ferreira 2022). A data matrix was built based on sequences available in the genbank database and obtained during the development of this study (Suppl. material 1). A list of ingroup and outgroup species, vouchers and GenBank accession numbers is given in Suppl. material 1.

### ﻿DNA extraction, amplification and sequencing

DNA of *Vanilla* species was extracted from fresh material according to a modified CTAB method (Doyle and Doyle 1987). The amplifications were carried out using 50 µL PCR volumes. Relaxation of the DNA strands was achieved by the addition of a 5M betaine solution to the PCR preparations. Primers of the nuclear ribosomal transcribed spacer region (ITS), including the 5.8S gene (Sun et al. 1994) were used for amplification and sequencing. *Taq* DNA polymerase was added to the PCR mixture at 80 °C following a 10 min period of denaturation at 99 °C in the thermocycler. Thirty-five cycles were run according to the following program: denaturation, 1 min, 94 °C; annealing, 45 sec, 64 °C; extension, 1 min, 72 °C; final extension, 5 min, 72 °C. Amplified PCR products were purified using GFX PCR columns (GE Health Care). Sequencing reactions were prepared using Big Dye 3.1 (ABI), purified PCR products and the same aforementioned primers. Samples were dehydrated and re-suspended with loading dye. Sequences were obtained using an Applied Biosystems automated sequencer model 3100. Sequence Navigator and Autoassembler (Applied Biosystems) software was used for sequence editing and assembly of complementary and overlapping sequences. DNA sequences were aligned using the BioEdit version 5.0.9 software (Hall 1999). The sequence alignment is available upon request from the first author.

### ﻿Phylogenetic analyses

A data matrix of ITS containing 43 taxa was used for phylogenetic analyses.

Maximum parsimony analysis (MP) was run with PAUP* version 4.0b5 (Swofford 2001) software. A heuristic search was conducted with 1000 replicates of random taxon entry additions, MULTREES option, and the tree bisection-reconnection (TBR) swapping algorithm, holding 10 trees per replicate and saving all the shortest trees. Support for clades was assessed using 1,000 bootstrap replicates (Felsenstein 1985). Bootstrap support (BS) values above 50% were calculated and mapped above the branches of the consensus tree. For bootstrap support levels, we considered bootstrap percentages of 50–70% as weak, 71–85% as moderate, and >85% as strong (Kress et al. 2002).

Maximum Likelihood (ML) analysis was run using the MEGA X tool (Kumar et al. 2018) with bootstrapping for 3,000 replicates. The analysis was based on the Kimura 2-parameter model (Kimura 1980). Initial trees for the heuristic search were obtained automatically by applying Neighbour-Joining and BioNJ algorithms to a matrix of pairwise distances estimated using the Maximum Composite Likelihood (MCL) approach, and then selecting the topology with the superior log likelihood value.

Bayesian Inference (BI) was conducted using the MrBayes program, version 3.1 (Ronquist and Huelsenbeck 2003). The optimal model of sequence evolution for each partition was selected using MEGA X (Kumar et al. 2018) and Bayesian Information Criterion (BIC). The software selected the HKY+G as the best evolution model for the ITS region. Four Markov chains were run simultaneously for three million generations, with parameters sampled every 100 generations. The consensus tree was calculated after removal of the first 3,000 trees, which were considered to be “burn-in”. Posterior probability (PP) values above 0.5 were calculated and mapped below the branches of the consensus tree.

## ﻿Results

### ﻿Taxonomic treatment

#### ﻿ Vanilla rupicola

Taxon classificationPlantaeAsparagalesOrchidaceae

Pansarin & E.L.F.Menezes sp. nov.

B3314350-8165-5577-BC91-0CF7A951AA26

urn:lsid:ipni.org:names:77320774-1

Figs 1 , 2

##### Type.

Brazil. Minas Gerais: Mun. Diamantina, Distrito de Sopa, Afloramento rochoso ca. 2 km de Morrinhos, 18°11'43"S, 43°43'18"W, 817 m, 29 November 2022, *E.R. Pansarin & E.L.F. Menezes 1561* (holotype: SPFR18105!).

*Vanillarupicola* differs from all Neotropical *Vanilla* species by its rupicolous habit, its reptant stem and its rounded leaves. The overall characteristics of *V.rupicola* resemble those of *V.appendiculata* Rolfe and *V.hartii* Rolfe. However, the remarkable new taxon (*V.rupicola*) is easily distinguishable from both related species by its leaves and flowers, which are smaller than those of *V.appendiculata* and larger than those of *V.hartii* (Table 1), its sessile leaves (vs. petiolate in *V.appendiculata* and *V.hartii*), and its papillose labellar protrusions (vs. finger-like in *V.appendiculata* and verrucose in *V.hartii*).

**Table 1.** Comparison of the morphological features of *Vanillarupicola* and of closely related species. Morphological data from *V.hartii* were obtained from Ferreira et al. (2020), while characteristics of *V.appendiculata* were obtained from Engels and Ferneda Rocha (2016)^a^ and Barona-Colmenares (2018)^b^.

Species characteristic	*Vanillarupicola*	*Vanillaappendiculata*	*Vanillahartii*
Habit	rupicolous	hemiepiphytic^a, b^	hemiepiphytic
Stem	reptant	scandent^a, b^	scandent
Leaves	3.2–9 × 2.8–5.2 cm	13–19 × 3.9–5.7 cm^a^	6.5–8 × 2.5–3.5 cm
		14–17.7 × 4.2–49 cm^b^	
Leaves	sessile	petiolate^a, b^	petiolate
Leaf blade	ovate to rounded	obovoid^a^	elliptic
		spatulate^b^	
Inflorescence	apical/lateral	apical^a, b^	lateral
Sepals	5.8–6.3 × 1–1.3 cm	6.6 × 0.7 cm^a^	4.9–5.3 × 0.8–1.1 cm
		7.5–7.8 × 0.8–1.1 cm^b^	
Petals	5.7–6.2 × 0.7–1.2 cm	6.5 × 0.6 cm^a^	4.9–5.1 × 0.7–0.9 cm
		7.6–7.7 × 0.7–0.8 cm^b^	
Labellum	5.6–6.3 × 3.2–3.5 cm	6.8 × 1.6 cm^a^	4.4–4.7 × 2.3–2.4 cm
		3.8 × 2.9 cm^b^	
Labellar protrusions	papillous	finger-like^b^	verrucose
Column	4.2–4.8 × 0.3–0.4 cm	5.4 × 0.2 cm^a^	3.8–4 × 0.2–0.3 cm
		6.1 × 0.3 cm^b^	

##### Description.

Rupicolous herbs up to 12 m in length. Roots axillary, 1.8–2.2 mm diam., yellowish-green, one per node. Stem reptant, flexuous, cylindrical, fleshy, glabrous, green to yellowish-green; internodes 40–130 × 3–13 mm. Leaves 3.2–9 × 2.8–5.2 cm, alternate, ovate to rounded, fleshy, glabrous, green to yellowish-green, apex acute to acuminate, sessile. Inflorescence 4.5–6 cm long, apical or axillary, racemose, with up to 10 flowers opening in succession; one flower opening each morning; bracts 5–9 × 3.5–7 mm progressively smaller toward the apex, alternate, triangular to ovate, coriaceous, adpressed to patent, concave, apex acute to acuminate. Flowers resupinate, whitish-green, pedicellate, abscission layer between perianth and ovary present; pedicel with ovary 2.8–3.2 × 0.40–0.52 cm, green, incurved, dilated to the apex, triangular in transverse section. Sepals 5.8–6.3 × 1–1.3 cm, free, oblanceolate, fleshy, slightly concave, pale green, margin entire, slightly incurved, apex acute to obtuse; dorsal sepal symmetric; lateral sepals asymmetric. Petals 5.7–6.2 × 0.7–1.2 cm, free, linear to slightly spatulate, asymmetric, membranous, pale green, apex acute to acuminate, adaxial surface with a longitudinal rib. Labellum 3-lobed, 5.6–6.3 × 3.2–3.5 cm, white, inner surface with longitudinal brown stripes, with a prominent central crest near the apex, and with a penicillate callus just below the anther; central crest ca. 4–5 mm wide, with yellow or white papillose protrusions arranged in five longitudinal lines near the apex; penicillate callus 5–6 × 5.2 mm, white; margins fused from the base to ca. ¾ of the column length forming a tubular nectar chamber; nectary chamber 2.2–2.6 cm in length; lateral lobes rounded, overlapping above the column, undulate margins; apical lobe rounded to emarginated, reflexed, undulate to slightly fimbriate. Column 4.2–4.8 × 0.3–0.4 cm, semi-cylindrical, slender, sinuous, white, attenuate base, dilated to the apex, with white-hyaline hairs close to the stigma, apex ending in a membranous ochrea; anther 4.9–5.1 × 3.9–4.2 mm, white, versatile; rostellum 3.8–4.1 × 3.9–4.0 mm, rectangular to trapezoidal, white. Fruits 9–13 × 1–1.8 cm, linear, tapering towards the apex, triangular in transverse section, green when immature. Seeds ovate, black.

**Figure 1.**
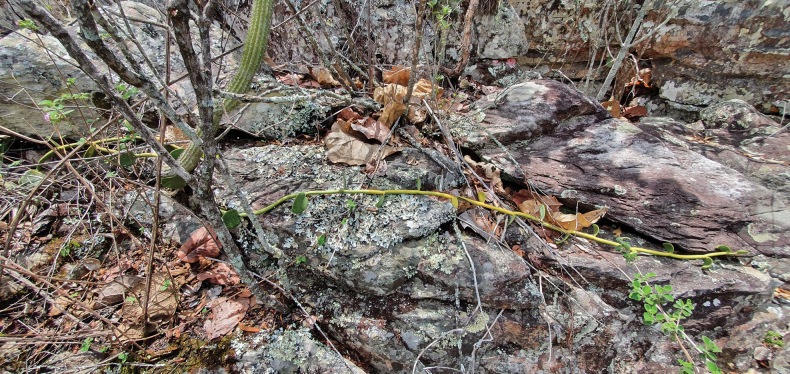
Habit of *Vanillarupicola* Pansarin & E.L.F. Menezes on the rock outcrops of the Espinhaço Range, Minas Gerais, Brazil. Note the creeping stem on the rock.

**Figure 2.**
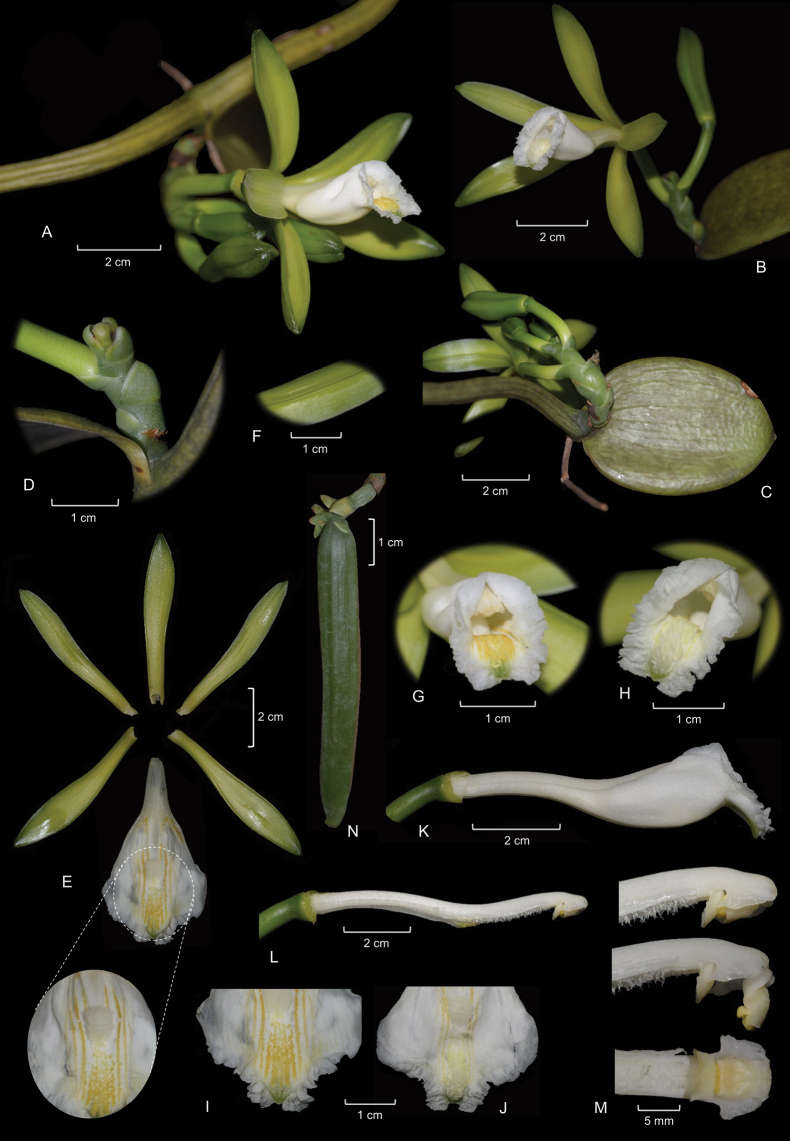
*Vanillarupicola* Pansarin & E.L.F. Menezes **A** part of a flowering plant showing the stem and a lateral inflorescence with a typical-colored flower **B** detail of an apical inflorescence of an albino plant. Note the white flower **C** leaf and inflorescence **D** detail of a raceme. Note the adpressed floral bracts **E** dissected perianth. The detail shows the penicillate callus and the central labellar crest **F** detail of the adaxial surface of a petal showing the longitudinal keel **G** detail of a flower with a typical color showing the apex of the labellum, the penicillate callus and anther **H** detail of an albino flower showing the apex of the labellum, the penicillate callus and anther. Note that the labellum apex is more projected than in the typically-colored flower **I** detail of the apex of the labellum of a typically-colored flower showing the yellowish longitudinal lines and the yellow projections **J** detail of the apex of the labellum of an albino flower showing the whitish projections **K** labellum in lateral view **L** column in lateral view **M** apex of the column: in lateral view with an articulated anther (above), in lateral view with a disarticulated anther (mid), and in abaxial view (below) **N** immature fruit.

##### Distribution and ecology.

The species has been reported for the campos rupestres vegetation of the Espinhaço Range of Minas Gerais (ERMG), municipality of Diamantina, Southeastern Brazil. In this locality, *Vanillarupicola* shows a reptant habit on rock outcrops and rooting in rock clefts. The elevation is from 800 to 1300 m a.s.l. The flowers produce a sweet fragrance perceptible during the hottest hours of the day. Each flower lasts ca. 12 hours.

##### Phenology.

*Vanillarupicola* has been collected with flowers from late September to early November. The fruits ripen from May to June.

##### Etymology.

The specific epithet (*rupicola*) refers to the rupicolous habit, uncommon among Neotropical *Vanilla*.

##### Conservation status.

*Vanillarupicola* is a rare species currently known to grow in a mountain-chain of Diamantina, in the ERMG. The populations found are composed of few specimens. According to the IUCN red list categories and criteria and guidelines, the species can tentatively be considered as Endangered (EN) due to its geographic range which is estimated to consist of 30 km^2^, and fewer than 250 individuals were recorded in the occurrence area.

##### Additional specimens examined (paratypes).

Brazil. Minas Gerais: Mun. Diamantina, Parque Nacional das Sempre Vivas, Próximo a Serra do Landim, 17°53'27,6"S, 43°45'51.3"W, 1293 m elev., 22 October 2019, *E.L.F. Menezes 133* (DIAM!). Município de Sopa. Estrada vicinal, 18°11'33.2"S, 43°47'07.5"W, 1210 m alt., 19 November 2022, *E.L.F. Menezes 752* (HDJF!); Distrito de Sopa, Afloramento rochoso ca. 2 km de Morrinhos, 18°11'43"S, 43°43'18"W, 817 m, 29 November 2022, *E.R. Pansarin & E.L.F. Menezes 1562* (BHCB!); Distrito de Sopa, Afloramento rochoso ca. 2 km de Morrinhos, 18°11'43"S, 43°43'18"W, 817 m, 29 November 2022, *E.R. Pansarin & E.L.F. Menezes 1563* (UEC!); Distrito de Sopa, Afloramento rochoso ca. 2 km de Morrinhos, 18°11'43"S, 43°43'18"W, 817 m, 29 November 2022, *E.R. Pansarin & E.L.F. Menezes 1564* (SP!); Distrito de Sopa, Afloramento rochoso ca. 2 km de Morrinhos, 18°11'43"S, 43°43'18"W, 817 m, 29 November 2022, *E.R. Pansarin & E.L.F. Menezes 1565* (LBMBP!).

##### Morphological affinities.

*Vanillarupicola* is easily recognized by its uncommon rupicolous habit, by its reptant stems, and by its sessile and rounded leaves. These characteristics differ significantly from those of the remaining non-membranaceous *Vanilla* species, which are characterized by their hemiepiphytic or more rarely epiphytic habit and by their elliptic to lanceolate leaves. Floral features suggest a close relationship between *V.rupicola* and some species currently recognized in the *V.planifolia* group and *V.trigonocarpa* group mainly regarding the type of appendages of the central crest of the labellum, and labellar color pattern, with longitudinal brown lines over a white background that converge at the entrance of the nectary. In addition, species of the *V.planifolia* group show a white penicillate callus just below the gynostemium apex. Among the members currently recognized in the *V.planifolia* group, our data suggest taxonomic affinities between *V.rupicola* and the Amazonian *V.appendiculata.* In fact, both species share characteristics that are uncommon among Neotropical *Vanilla*, such as the production of apical inflorescences and ovate to obovoid leaf blades. The inflorescences of members of the *V.planifolia* group are lateral, and their leaves are elliptical to lanceolate. In addition, *V.rupicola* also shares some floral features with the Amazonian *V.hartii* (currently included in the *V.trigonocarpa* group), such as the lip lobes with undulate margins, labellar protrusions arranged on five longitudinal lines near the apex, and brown lines converging at the nectar chamber. The main differences between *V.rupicola* and related species (*V.appendiculata* and *V.hartii*) are summarized in Table 1.

### ﻿Phylogenetic relationships

**Figure 3.**
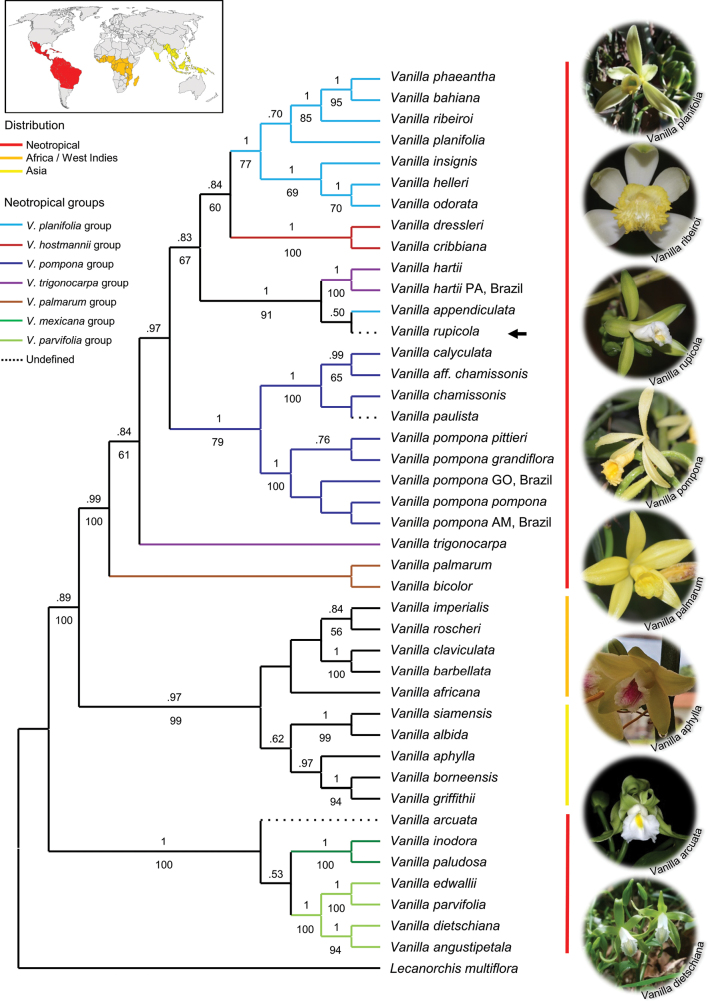
Maximum Likelihood analysis of *Vanilla* (Orchidaceae) based on ITS (nrDNA). Bootstrap values (%) >50 obtained by maximum parsimony analysis (MP) are given below the branches, while posterior probabilities values > 0.5 (BI) are given above branches. Vertical colored bars refer to the geographic distribution of *Vanilla*. The black arrow indicates the position of *V.rupicola* among the Neotropical thick-leafed *Vanilla*. The colored branches in the cladogram refer to Neotropical *Vanilla* groups according to the infrageneric classification presented in Soto-Arenas and Cribb (2010). AM = Amazonas, GO = Goiás, PA = Pará.

Phylogenies obtained by analysis of the ITS1-5.8S-ITS2 region using distinct methods (BI, ML and MP) yielded trees with congruent topologies (ML (Fig. 3) is shown). In all analyses, the Neotropical *Vanilla* with reticulate-veined leaves (PP 1, BS 100%; Fig. 3) was recovered as sister to a large clade (PP .89, BS 100%) with two subclades: an Old-World/Caribbean clade (PP .97, BS 99%), and a well-supported clade including the remaining Neotropical *Vanilla* (PP .99, BS 100%; Fig. 3). Among the Neotropical reticulate-veined *Vanilla*, *V.arcuata* Pansarin & Miranda emerged as sister -without support- to a clade containing two sister groups, the *Vanillamexicana* group, (*V.inodora* Schiede/*V.paludosa* Pansarin, J.M. Aguiar & A.C. Ferreira; PP 1, BS 100%) and the *V.parvifolia* group (*V.diestschiana* Edwall, *V.angustipetala* Schltr., *V.edwallii* Hoehne and *V.parvifolia* Barb. Rodr.; PP 1, BS 100%). Two well-supported clades were recovered within the latter group: *V.diestschiana*/*V.angustipetala* (PP 1, BS 94%), and *V.edwallii*/*V.parvifolia* (PP 1, BS 100%; Fig. 3). Within the Old-World/Caribbean *Vanilla*, the phylogenetic analyses (BI, ML and MP) recovered two poorly supported subclades: an Asian clade, and an African/Caribbean clade (Fig. 3). In the Asian *Vanilla*, the clade including *V.siamensis* Rolfe ex Downie and *V.albida* Blume (PP 1, BS 99%) emerged as sister to a clade comprising *V.aphylla* Blume/(*V.borneensis* Rolfe/*V.griffithii* Rchb. f.). In the African/Caribbean clade, *V.africana* Lindl. was nested as sister to two subclades: a West Indies clade that included *V.claviculata* Sw. and *V.barbellata* Rchb. f. (PP 1, BS 100%), and an African clade containing *V.imperialis* Kraenzl. /*V.roscheri* Rchb. f. (PP .84, BS 56%; Fig. 3). Within the thick-leaved Neotropical *Vanilla* (PP .99, BS 100%), species currently recognized among the *V.palmarum* group, i.e. the epiphytes *V.palmarum* and *V.bicolor* Lindl., emerged as sisters to a large clade whose species are mostly hemiepiphytes or nomadic vines (PP .84, BS 61%). Within the hemiepiphytic clade, the Amazonian *V.trigonocarpa* Hoehne emerged as sister to a clade including the remaining Neotropical taxa. The latter showed two subclades: one including the members of the *V.pompona* group (*V.pompona* Schiede, *V.calyculata* Schltr., and *V.chamissonis* Klotzsch, besides *V.paulista* Fraga & Pansarin (PP 1, BS 79%)), and the other a large and predominantly Amazonian clade that contained the remaining Neotropical taxa (Fig. 3). In the latter, a strongly supported clade (PP 1, BS 91%) including *V.hartii* (V. *rupicola*/*V.appendiculata*) emerged as sister to a large clade in which the members of the *V.hostmanii* group, *V.dressleri* Soto Arenas/*V.cribbiana* Soto Arenas (PP 1, BS 100%) were recovered as sister to a large clade that included two subclades: *V.insignis* Ames/(*V.odorata* C. Presl/*V.helleri* A.D. Hawkes) plus *V.planifolia*/(*V.ribeiroi* Hoehne/(*V.phaeantha* Rchb. f./*V.bahiana* Hoehne)) with moderate support (PP 1, BS 77%; Fig. 3).

## ﻿Discussion

Neotropical *Vanilla* species are represented by two distinct lineages, one including representatives with membranous leaves and the other including species with non-membranous, usually fleshy, leaves. Our results strongly agree with previous phylogenetic inferences based on the sequencing of the cpDNA (Bouetard et al. 2010) and nrDNA (Pansarin and Ferreira 2022) regions. The membranous reticulate-veined *Vanilla* (i.e. subgenus Vanilla; Soto-Arenas and Cribb 2010) includes members of two groups, i.e.: *V.mexicana* group and *V.parvifolia* (non *V.parviflora*) group. In fact, our data matrix including rare and endemic species from Brazil recovered both groups proposed for the subgenus Vanilla. However, although *V.arcuata* is a member of the membranaceous-leafed *Vanilla* (Pansarin and Miranda 2016), our data suggest further studies are needed in order to better determine the circumscription of infrageneric groups proposed by Soto-Arenas and Cribb (2010), as this Brazilian species emerges in a basal position within this Neotropical subgenus (Fig. 3).

Among the non-membranaceous *Vanilla* taxa, the basal taxa *V.palmarum* and *V.bicolor*, both occurring as epiphytes on palms (e.g. Soto-Arenas and Cribb 2010; Van Dam et al. 2010, E.R. Pansarin, pers. obs.), emerge as sisters to the hemiepiphytic and thick-leafed groups. Among the groups proposed by Soto-Arenas and Cribb (2010), the *V.pompona* group is recovered as monophyletic (*V.paulista* included). Within this clade, *V.aff.chamissonis*, which is found in semideciduous forests and Cerrado areas of Brazil, is more related to *Vanillacalyculata* (= *V.columbiana* Rolfe, Karremans et al. 2020) than to *Vanillachamissonis*, which occurs along the Atlantic coast. Further studies involving members of this *Vanilla* group are needed in order to better understand their species boundaries. According to the classification of Soto-Arenas and Cribb (2010), *V.appendiculata* is a member of the *V.planifolia* group, while *V.hartii* has been placed in the *V.trigonocarpa* group. Our data suggest that realignment is necessary because *V.trigonocarpa* emerges in a basal position within the non-membranous hemiepiphyte *Vanilla*, while *V.hartii* emerges among the most derived clades in the genus. Indeed, the clade containing *V.hartii*, *V.rupicola* and *V.appendiculata* emerges as sister to the *V.hostmanii* group. Consequently, both the *V.trigonocarpa* group and *V.planifolia* group are polyphyletic (Soto-Arenas and Cribb 2010). According to the current *Vanilla* classification, *V.rupicola* emerges in an unrecognized group. As detailed in the results section (see Morphological Affinities and Table 1), *V.rupicola* shares a number of characteristics with *V.hartii* and *V.appendiculata*, mainly with regard to leaf shape, inflorescence position (i.e. apical, as in *V.appendiculata*), the type of appendages of the central crest of the labellum, and labellar color pattern (Ferreira et al. 2020; Engels and Ferneda Rocha 2016; Barona-Colmenares 2018).

The rupiculous habit of *V.rupicola* is unique among the members of the non-membranaceous Neotropical *Vanilla*. The emergence of a rupicolous species within an entirely hemiepiphytic clade appears to be a result of evolutionary convergence (reversion), as some leafless Old-World species grow on rocks (e.g. Andriamihaja et al. 2020). Despite its rarity in Neotropical *Vanilla*, the rupicolous habit is widespread among Angiosperms distributed in the Espinhaço Range (e.g. Barros 1987; Azevedo and Van Den Berg 2007). Indeed, Neotropical *Vanilla* species are diverse and widespread throughout the Atlantic Forest and the Amazon Biome (Hoehne 1945). Based on the evidence that *V.rupicola* emerges in an essentially Amazonian clade, it seems plausible that the ancestor of this new taxon derived from an Amazonian ancestor adapted to the environmental conditions of the Espinhaço Range and evolved in this particular environment. Nowadays, there is consensus about the evolution of the Neotropical Biomes. During the Paleogene, the ancient Amazon Forest and Atlantic Forest were spatially interconnected and continuous (Morley 2000). The Espinhaço refuges evolved between the two Biomes as a consequence of extreme environmental conditions and climatic fluctuations during the Tertiary and Quaternary periods (Zappi 2008). Their unique geomorphological and climatic conditions resulted in a huge number of endemisms (Rapini et al. 2008; Zappi 2008), whose exclusive diversity is the consequence of a long process of evolution in this singular environment (Silva et al. 2008). Many orchids occur exclusively in the Espinhaço Range of Minas Gerais (e.g. Barros 1987; Azevedo and Van Den Berg 2007; Batista et al. 2016; Salazar et al. 2019). Here, an endemic *Vanilla* from the ERMG is reported for the first time. This is not surprising because Brazil, with more than 38 species, is the center of diversification for Neotropical *Vanilla* (Pansarin 2019). Furthermore, five species are endemic to the Brazilian Biomes (Hoehne 1945; Pansarin 2010; Pansarin and Miranda 2016; Fraga et al. 2017). Therefore, knowledge about the taxonomy and phylogeny of Brazilian *Vanilla* is fundamental in order to understand the evolution and natural history of this Pantropical orchid genus (Pansarin et al. 2012).

## Supplementary Material

XML Treatment for Vanilla rupicola

## ﻿Additional information

### Author ORCIDs

Emerson Ricardo Pansarin https://orcid.org/0000-0002-0355-8363

Euler da Luz Fernandes Menezes https://orcid.org/0000-0002-1861-9552

### Data availability

All of the data that support the findings of this study are available in the main text or Supplementary Information.

## Supplementary materials

Supplementary material 1 Species of *Vanilla* included in the molecular study, their locations, vouchers and GenBank accession numbers Data type docx. file Explanation note VAN = Vanilla germplasm bank, LBMBP Orchid House, Department of Biology, FFCLRP-USP, University of São Paulo, Brazil available at https://www.lbmbplab.net/vanillacollection. https://binary.pensoft.net/file/861414This dataset is made available under the Open Database License (http://opendatacommons.org/licenses/odbl/1.0/). The Open Database License (ODbL) is a license agreement intended to allow users to freely share, modify, and use this Dataset while maintaining this same freedom for others, provided that the original source and author(s) are credited.Emerson Ricardo Pansarin, Euler da Luz Fernandes Menezes
