# Supplementary material for: ﻿A new remarkable Vanilla Mill. (Orchidaceae) species endemic to the Espinhaço Range, Brazil: its phylogenetic position and evolutionary relationships among Neotropical congeners
Source: PhytoKeys. 2023 Jun 8;227:151–65. doi: 10.3897/phytokeys.227.101963 (PMC10273138; doi:10.3897/phytokeys.227.101963)
Supplement: Supplementary material 1 — Species of Vanilla included in the molecular study, their locations, vouchers and GenBank accession numbers [file phytokeys-227-151_article-101963__-s001.docx]

**Table S1.** Species of *Vanilla* included in the molecular study, their locations, vouchers and GenBank accession numbers

| Species | Location | Voucher | GenBank accessions |
| --- | --- | --- | --- |
| *Lecanorchis multiflora* J.J. Sm. |  | Cameron 1015 | MN902000 |
| *Vanilla africana* Lindl. |  | Chase O-584 (K) | FJ425834 |
| *Vanilla albida* Blume | Indonesia | VAN003 |  |
| *Vanilla angustipetala* Schltr. | São Paulo, Brazil | Pansarin & Miranda s.n. |  |
| *Vanilla appendiculata* Rolfe | Mato Grosso, Brazil | VAN112 |  |
| *Vanilla aphylla* Blume |  | - | AF151006 |
| *Vanilla arcuata* Pansarin & Miranda | São Paulo, Brazil | Pansarin & Miranda 1514 (LBMBP) |  |
| *Vanilla bahiana* Hoehne | Espírito Santo, Brazil | VAN 077 |  |
| *Vanilla barbellata* Rchb.f. |  | Chase 591 (K) | MN902001 |
| *Vanilla bicolor* Lindl. | Amazonas, Brazil | VAN 049 |  |
| *Vanilla borneensis* Rolfe |  | VAR038 | MH777722 |
| *Vanilla calyculata* Schltr. | HD: Lizapa | Linares 8531 | MN902004 |
| *Vanilla chamissonis* Klotzsch | São Paulo, Brazil | VAN 079 |  |
| *Vanilla aff. chamissonis* Klotzsch | São Paulo, Brazil | VAN 046 |  |
| *Vanilla claviculata* Sw. |  | Rostgaard s.n. | MN902007 |
| *Vanilla cribbiana* Soto Arenas | MX: Chiapas, Lacanjá-Chanzayab | Soto 8439 (AMO) | MN902009 |
| *Vanilla dressleri* Soto Arenas | CR: Punta Arenas, Cañaza, F. Don Andrés | Byrd I-A-6 | MN902011 |
| *Vanilla edwallii* Hoehne | São Paulo, Brazil | Pansarin 840 (UEC) | EU498165 |
| *Vanilla dietschiana* Edwall | São Paulo, Brazil | Pansarin and Pansarin 1278 (SPFR) |  |
| *Vanilla griffithii* Rchb.f. |  | VAR033 | MH777745 |
| *Vanilla hartii* Rolfe | Pará, Brazil | VAN 062 |  |
| *Vanilla hartii* Rolfe | CR: Puntarenas, Sándalo, Quebrada Terrones | Byrd II-F-1 | MN902017 |
| *Vanilla helleri* A.D. Walkes | CR: Punta Arenas, Cañaza-Sándalo | Byrd II-D-5 | MN902019 |
| *Vanilla imperialis* Kraenzl. |  | Chase O-587 (K) | FJ425830 |
| *Vanilla inodora* Schiede | MX: Jalisco, El Tuito | Soto 8626 (AMO) | MN902022 |
| *Vanilla insignis* Ames | MX: Campeche, Calakmul | Soto 7668 (AMO) | MN902023 |
| *Vanilla odorata* C. Presl | MX: Chiapas, Chajul | Soto 8356 (AMO) | MN902028 |
| *Vanilla parvifolia* Barb. Rodr. | São Paulo, Brazil | Pansarin s.n. (LBMBP) |  |
| *Vanilla phaeantha* Rchb.f. | PMA: Veraguas, El Higo | Soto 9920 (AMO) | MN902035 |
| *Vanilla palmarum* (Salzm. *ex* Lindl.) Lindl. | Amazonas, Brazil | Pansarin 1168 (INPA) |  |
| *Vanilla paludosa* Pansarin, J.M. Aguiar & A.C. Ferreira | São Paulo, Brazil | Pansarin, Aguiar & Ferreira 1361 (SPFR) |  |
| *Vanilla paulista* Fraga & Pansarin | São Paulo, Brazil | Pansarin 727 (UEC) | EU498163 |
| *Vanilla planifolia* Andrews | MX: Chiapas, Chajul | Soto 8355 | MN902039 |
| *Vanilla pompona* Schiede | Amazonas, Brazil | Pansarin 1167 (INPA) | EU498164 |
| *Vanilla pompona* Schiede | Goiás, Brazil | VAN 006 |  |
| *Vanilla pompona* subsp. *grandiflora* (Lindl.) Soto Arenas | GYF: Sinnamary, St. Elie | Veyret 1 | MN902049 |
| *Vanilla pompona* subsp. *pittieri* (Schltr.) Dressler | CR: Punta Arenas: Pto. Jiménez, F. La Pajuita | Byrd I-A-2 | MN902059 |
| *Vanilla pompona* Schiede subsp. *pompona* | MX: Nayarit, El Cuarenteño-El Cora | Soto 8614 (AMO) | MN902053 |
| *Vanilla ribeiroi* Hoehne | Pará, Brazil | VAN 031 |  |
| *Vanilla roscheri* Rchb.f. |  | Chase O-540 (K) | FJ425840 |
| *Vanilla rupicola* Pansarin & Menezes | Minas Gerais, Brazil | Pansarin & Menezes 1551 (SPFR) |  |
| *Vanilla siamensis* Rolfe ex Downie |  | Chase O-540 (K) | JF825978 |
| *Vanilla trigonocarpa* Hoehne | CR: Puntarenas, Piró | Byrd I-E-5 | MN902066 |

VAN = *Vanilla* germplasm bank, LBMBP Orchid House, Department of Biology, FFCLRP-USP, University of São Paulo, Brazil available at <https://www.lbmbplab.net/vanillacollection>.
